# Regulation of Signaling at Regions of Cell-Cell Contact by Endoplasmic Reticulum-Bound Protein-Tyrosine Phosphatase 1B

**DOI:** 10.1371/journal.pone.0036633

**Published:** 2012-05-24

**Authors:** Fawaz G. Haj, Ola Sabet, Ali Kinkhabwala, Sabine Wimmer-Kleikamp, Vassilis Roukos, Hong-Mei Han, Markus Grabenbauer, Martin Bierbaum, Claude Antony, Benjamin G. Neel, Philippe I. Bastiaens

**Affiliations:** 1 Beth Israel Deaconess Medical Center, Harvard University, Boston, Massachusetts, United States of America; 2 Nutrition Department, University of California Davis, Davis, California, United States of America; 3 Department of Systemic Cell Biology, Max Planck Institute of Molecular Physiology, Dortmund, Germany; 4 European Molecular Biology Laboratories, Heidelberg, Germany; 5 Campbell Family Cancer Research Institute, Ontario Cancer Institute, Princess Margaret Hospital, University Health Network, and Department of Medical Biophysics, University of Toronto, Toronto, Ontario, Canada; Ecole Polytechnique Federale de Lausanne, Switzerland

## Abstract

Protein-tyrosine phosphatase 1B (PTP1B) is a ubiquitously expressed PTP that is anchored to the endoplasmic reticulum (ER). PTP1B dephosphorylates activated receptor tyrosine kinases after endocytosis, as they transit past the ER. However, PTP1B also can access some plasma membrane (PM)-bound substrates at points of cell-cell contact. To explore how PTP1B interacts with such substrates, we utilized quantitative cellular imaging approaches and mathematical modeling of protein mobility. We find that the ER network comes in close proximity to the PM at apparently specialized regions of cell-cell contact, enabling PTP1B to engage substrate(s) at these sites. Studies using PTP1B mutants show that the ER anchor plays an important role in restricting its interactions with PM substrates mainly to regions of cell-cell contact. In addition, treatment with PTP1B inhibitor leads to increased tyrosine phosphorylation of EphA2, a PTP1B substrate, specifically at regions of cell-cell contact. Collectively, our results identify PM-proximal sub-regions of the ER as important sites of cellular signaling regulation by PTP1B.

## Introduction

The ubiquitously expressed non-receptor protein-tyrosine phosphatase (PTP1B) plays an important role in regulating diverse cellular signaling pathways, including those initiated by receptor tyrosine kinases (RTKs), cytokine receptors, integrins and cadherins [1]. Major insights into the physiological role of PTP1B were gained through the generation of knockout (KO) mice, which showed that PTP1B is a critical regulator of insulin sensitivity and energy balance *in vivo* [2], [3], [4], [5]. However, other functions for PTP1B have also been suggested, including roles in regulating cell-matrix [6] and cell-cell [7], [8], [9] interactions. Given the salutary metabolic effects of PTP1B deletion, it has emerged as a potential target for anti-diabetic and anti-obesity drug development [10], [11]. Consequently, it is important to understand its mechanism of action in detail.

PTP1B is anchored to the cytosolic face of the endoplasmic reticulum (ER) via a hydrophobic C-terminal targeting sequence [12], [13], which constrains its access to key substrates. Consistent with this localization, PTP1B dephosphorylates the activated epidermal growth factor receptor (EGFR), platelet-derived growth factor receptor (PDGFR) and insulin receptor (IR) only after endocytosis, as they transit past the ER [14], [15], [16]. PTP1B activity also is spatially regulated in the cell, thus creating distinct microenvironments that enable RTK signal propagation, followed by signal termination [17]. Recently, PTP1B has been identified as a potential regulator of RTK endocytosis. Eden *et al.* reported that PTP1B-EGFR interaction occurs through direct membrane contact between multivesicular bodies (MVB) and the ER, with PTP1B activity promoting the sequestration of EGFR to MVB internal vesicles [18]. Consistent with these findings, Stuible *et al.* identified the endosomal protein STAM2, which regulates sorting of activated RTKs for degradation, as a PTP1B substrate [19]. Collectively, these studies reveal that PTP1B is a major regulator of RTK endocytosis and signaling.

Although activated RTKs gain access to PTP1B only after endocytosis, PTP1B also can interact with some plasma membrane (PM)-bound substrates [20], [21]. For example, PTP1B targets forming cell-matrix adhesion contacts and contributes to the stabilization of focal adhesions. This process appears to involve dynamic extension of the ER via a microtubule-dependent process [6]. PTP1B also can access substrates at points of cell-cell contact [7], [8], [9], although how these interactions are regulated remains largely unexplored. In the present study, we assess the dynamics of PTP1B mobility and investigate its spatial-temporal regulation of signaling at regions of cell-cell contact. Through the combined use of PTP1B mutants, advanced cell imaging and mathematical modeling, we show that ER-anchored PTP1B can reach PM-localized substrates, but only at regions of cell-cell contact. These studies strongly suggest that the ER is structured and polarized towards cell-cell junctions, and identify these PM-proximal sub-regions of the ER as important sites of cellular signaling regulation by PTP1B.

## Results

### PTP1B Substrate-trapping Mutant Localizes to Regions of Cell-cell Contact

**Figure 1:**
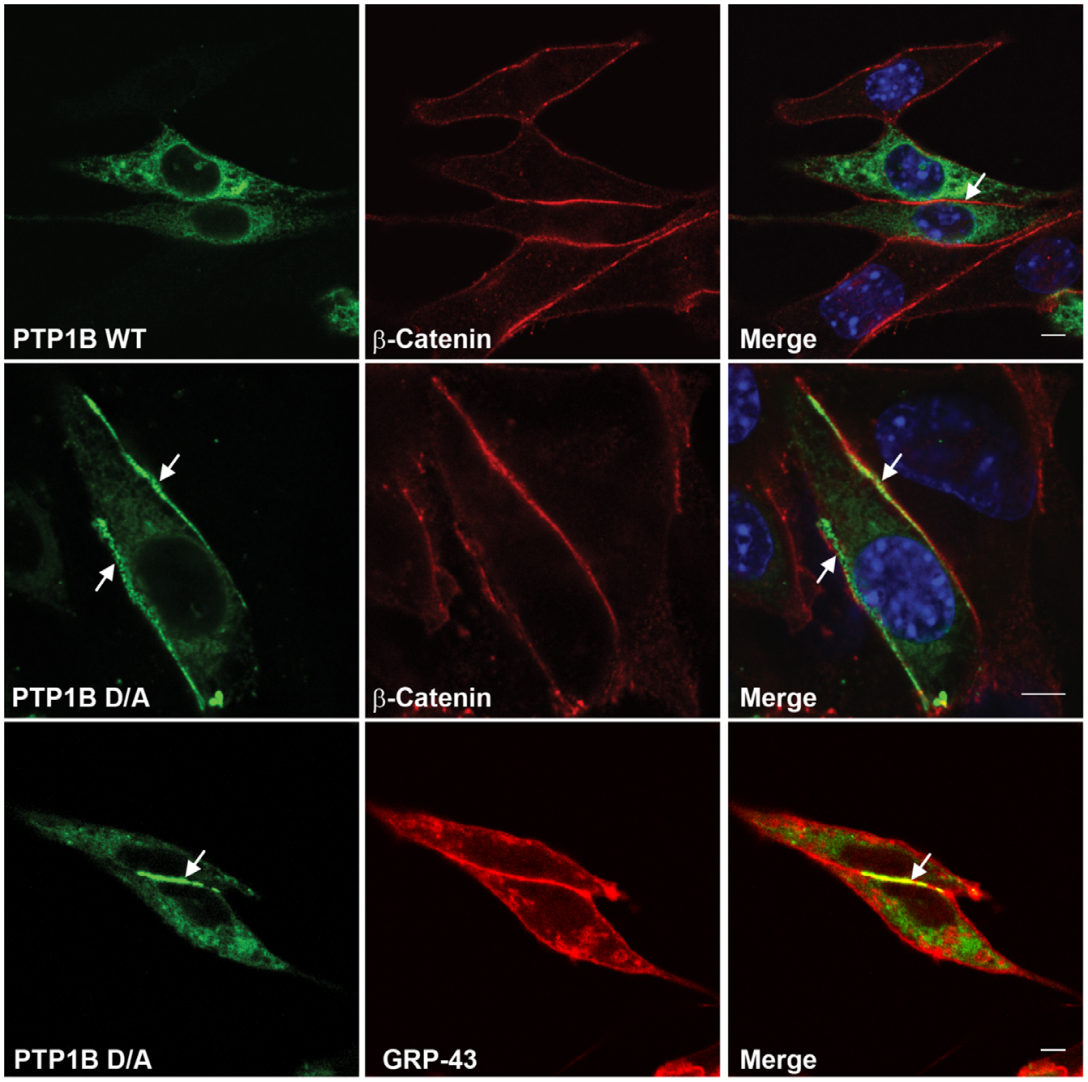
PTP1B localizes to regions of cell-cell contact. Randomly growing PTP1B-null fibroblasts transiently expressing PTP1B WT-GFP or PTP1B D/A-GFP were fixed and co-stained with β-catenin antibodies and DAPI (top and middle panels). The bottom panel shows fibroblasts co-expressing PTP1B D/A-GFP and the plasma membrane marker Grp43-mCherry in live cells. Regions of cell-cell contact are indicated with arrows. Scale bars correspond to 5 µm.

We transiently expressed green fluorescent protein (GFP)-tagged wild type (WT) PTP1B and its “substrate-trapping” mutant D181A (D/A), which retains substrate-binding but is catalytically impaired [22], in PTP1B-null fibroblasts, and monitored their sub-cellular localization using confocal microscopy (Fig. 1). Consistent with previous reports [12], [13], each localized to the ER network. However, PTP1B D/A, but not PTP1B WT, also accumulated at regions of cell-cell contact, labeled using anti-β catenin antibodies (Fig. 1). Studies using another PM marker, Cherry-tagged G protein-coupled receptor 43 (Grp43-Cherry), confirmed that PTP1B D/A accumulated at regions of cell-cell contact in live cells (Fig. 1, lower panel). Quantification of fluorescence intensity showed that about 10–15% of total cellular PTP1B D/A was found at cell-cell contacts. Similar findings were obtained using Cos-7, MDCK, 293T cells and 3T3-L1 preadipocytes (data not shown).

### The ER Extends to Regions Proximal to PM at Cell-cell Contacts

**Figure 2:**
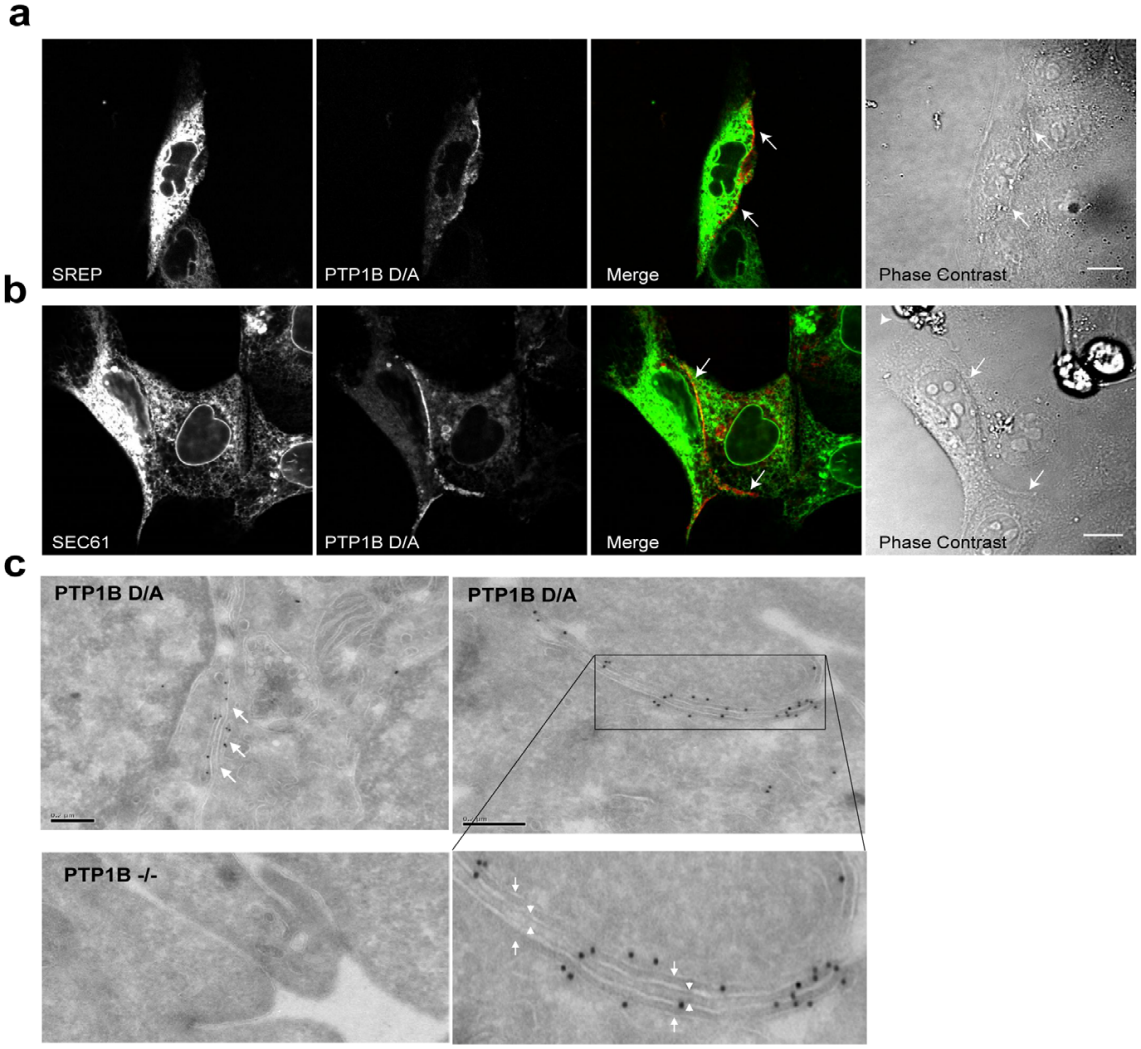
The endoplasmic reticulum lies in close proximity to the plasma membrane at regions of cell-cell contact. (**a**) MDCK cells co-expressing PTP1B D/A-mCherry and a general marker for the endoplasmic reticulum, stress-related ER protein (SREP-YFP); or (**b**) PTP1B D/A-mCherry Sec61-YFP, a marker for the rough ER. Arrows indicate region of cell-cell contact. Scale bars correspond to 10 µm. (**c**) Immunogold electron microscopy shows PTP1B D/A localization in the ER (arrows). No significant labeling was detected in PTP1B-null cells (left bottom image). Significant immunogold labeling was also detected at regions of cell-cell contact (right image). The boxed area, which is magnified below, shows the region of cell-cell contact that reveals labeling at the ER (arrows) proximal to the PM (arrowheads). Also see Fig. S2. Scale bars correspond to 0.2 µm.

These observations raised the question of how an ER-bound PTP might access substrates on or near the PM at regions of cell-cell contact. In platelets, PTP1B can be cleaved by calpain to release an active cytosolic fragment [23]. However, serial confocal images of tissue culture cells expressing PTP1B D/A-GFP, acquired at successive focal planes, revealed a “honeycomb” pattern at regions of cell-cell contact (Fig. S1a) which is characteristic of the reticular structure of the ER and consistent with its extension to these regions. Experiments in which PTP1B D/A was co-expressed in MDCK cells with the general ER marker, stress-related ER protein (SREP), confirmed that the ER extends out to the PM at regions of cell-cell contact (Fig. 2a, arrows). Comparable results were obtained when PTP1B D/A was co-expressed with a marker (Sec61) for rough ER (Fig. 2b). Exposure of cells to pervanadate, which oxidizes the essential cysteinyl residue found at the catalytic center of PTPs [24], abolished the localization of PTP1B D/A at cell-cell contacts without altering its ER localization (or that of PTP1B WT in the ER) (Fig. S1b). The latter findings indicate that PTP1B D/A enrichment at regions of cell-cell contact is mediated by interactions with one or more substrate(s).

Electron microscopy using immunogold-coupled anti-human PTP1B (FG6) antibodies revealed labeling of PTP1B D/A in the ER, providing direct evidence that PTP1B resides in this compartment (Fig. 2c, arrows, left image). Prominent labeling also was detected at regions of cell-cell contact (Fig. 2c, right image and inset), where four adjacent membranes were evident: two inner “thick” membranes (arrowheads) corresponding to the PMs of adjacent cells and two flanking “thinner” membranes (arrows), corresponding to the ER. The few gold particles that appeared to label the PM probably reflect the antigen-gold particle linker distance: because PTP1B should be separated from the gold particle by the distance of the antibody molecule in three dimensions [25], [26], the particle can lie directly over PTP1B or up to 10 nm away [26]. No significant labeling was detected in PTP1B-null fibroblasts (Fig. 2c), confirming the specificity of the immunogold labeling. To ask whether the observed proximity of ER to PM at regions of cell-cell contact is caused by PTP1B D/A expression, we performed routine EM and high resolution cryo-EM on vitreous sections from HeLa cells. These studies confirmed that the ER is in close proximity to the PM at regions of cell-cell contact even in the absence of PTP1B D/A (Fig. S2). Collectively, these studies show that the ER is in close proximity to the PM at regions of cell-cell contact and that ER-anchored PTP1B engages substrate(s) at these locations.

### PTP1B Mobility on ER Membranes

**Figure 3:**
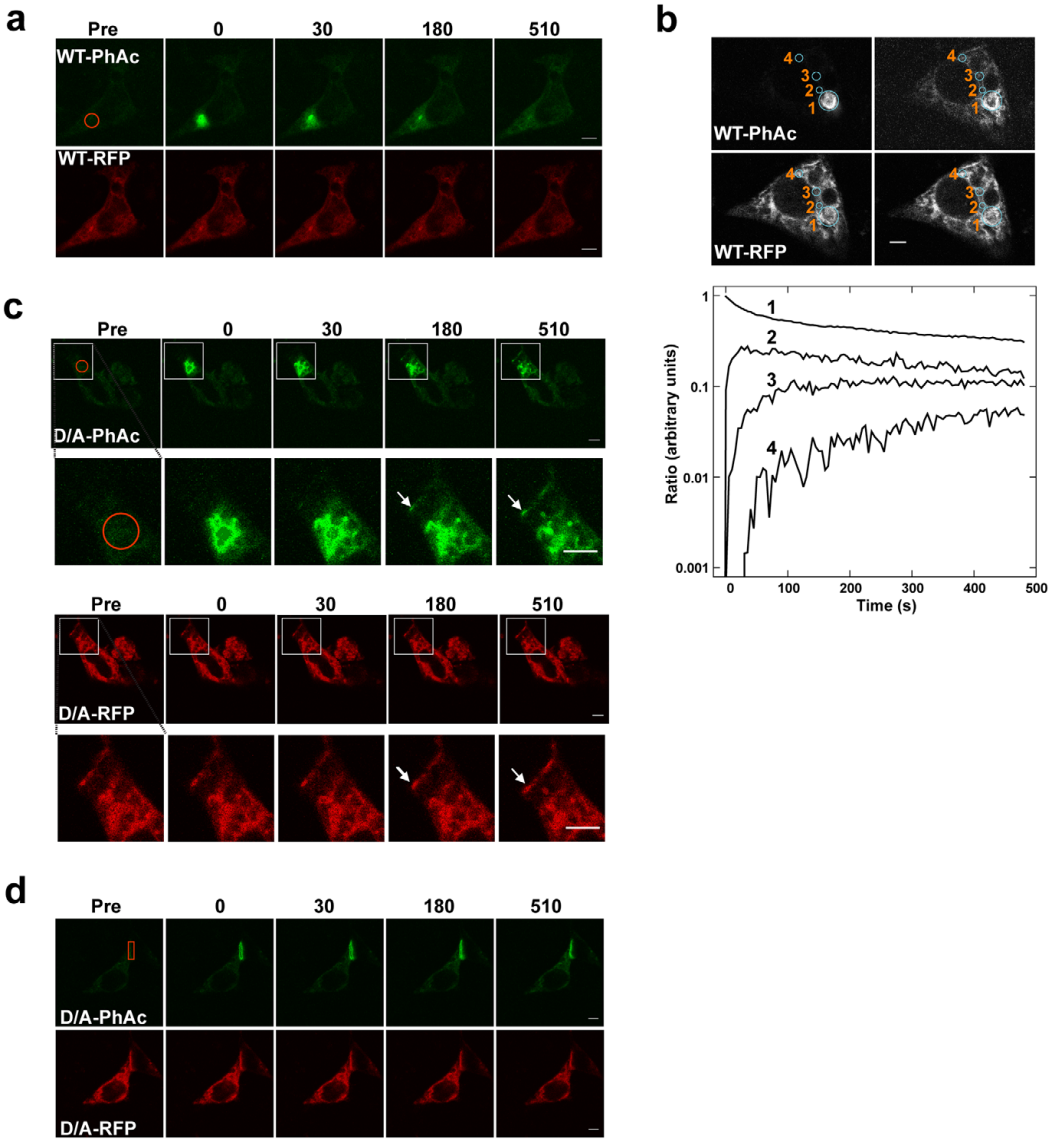
Diffusive motion of photoactivated PTP1B on the ER. (**a**) PTP1B-null fibroblasts co-expressing PTP1B WT-PhAc (upper panel) and PTP1B WT-RFP (lower panel) were photoactivated in the ER (region indicated with a circle), and the photoactivated pool was followed for the indicated times. (**b**) Cells were photoactivated at a region of interest (marked “1”), and PTP1B fluorescence intensity at this and distal regions (marked “2–4”) was quantified. The lower panel represents the change in fluorescence ratio (WT-PhAc/WT-RFP) at the four highlighted regions over time. (**c**) Cells co-expressing PTP1B D/A-PhAc and PTP1B D/A-RFP were photoactivated in the ER (circle). For each set of figures, the region in the boxed area of the top panel is shown magnified in the lower panel, with an arrow indicating the PM-proximal ER at regions of cell-cell contact (note that the adjacent cell is not transfected). (**d**) Cells co-expressing PTP1B D/A-PhAc and PTP1B D/A-RFP were photoactivated at regions of cell-cell contact (rectangle), revealing slower mobility compared to PTP1B D/A in the ER. Scale bars correspond to 5 µm.

To determine how an ER-bound phosphatase accesses PM substrate(s) at regions of cell-cell contact, we examined the mobility of PTP1B fused to photoactivatable GFP (PhAc), which can be used to mark a population of molecules in a region of interest and track them over time in live cells [27]. PTP1B WT-PhAc and PTP1B D/A-PhAc were transiently co-expressed in PTP1B-null cells with the corresponding RFP-tagged PTP1B, which served as a general ER marker and helped to identify transfected cells. Prior to photoactivation, no significant fluorescence attributable to PTP1B-PhAc (excitation at 488 nm) was detected (Fig. 3). Photoactivation (excitation at 413 nm) of selected regions resulted in pools of PTP1B WT or PTP1B D/A that became highly fluorescent upon excitation at 488 nm. Continuous imaging of the photoactivated pools revealed rapid, non-directional movement of PTP1B WT throughout the ER (Fig. 3a), with fluorescence intensity increasing gradually at regions distal from the photoactivation site (Fig. 3b; note intensity gain in distal region marked “4”). PTP1B D/A-PhAc (but not PTP1B WT-PhAc) that was initially photoactivated in the ER (away from sites of cell-cell contact) became detectable at regions of cell-cell contact, indicating that the ER is contiguous with these areas and is the source of PM-proximal PTP1B (Fig. 3c). By contrast, PTP1B D/A that was photoactivated at regions of cell-cell contact (rectangle) diffused away more slowly compared with pools activated in the ER (Fig. 3d; see below for detailed discussion of this apparent difference in speed). Thus, the movement of PTP1B is consistent with non-directional diffusion throughout the ER. Moreover, these studies show that the ER network is continuous, but lies proximal to the PM only at regions of cell-cell contact.

### PTP1B Mobility is Affected by Transient Interactions

**Figure 4:**
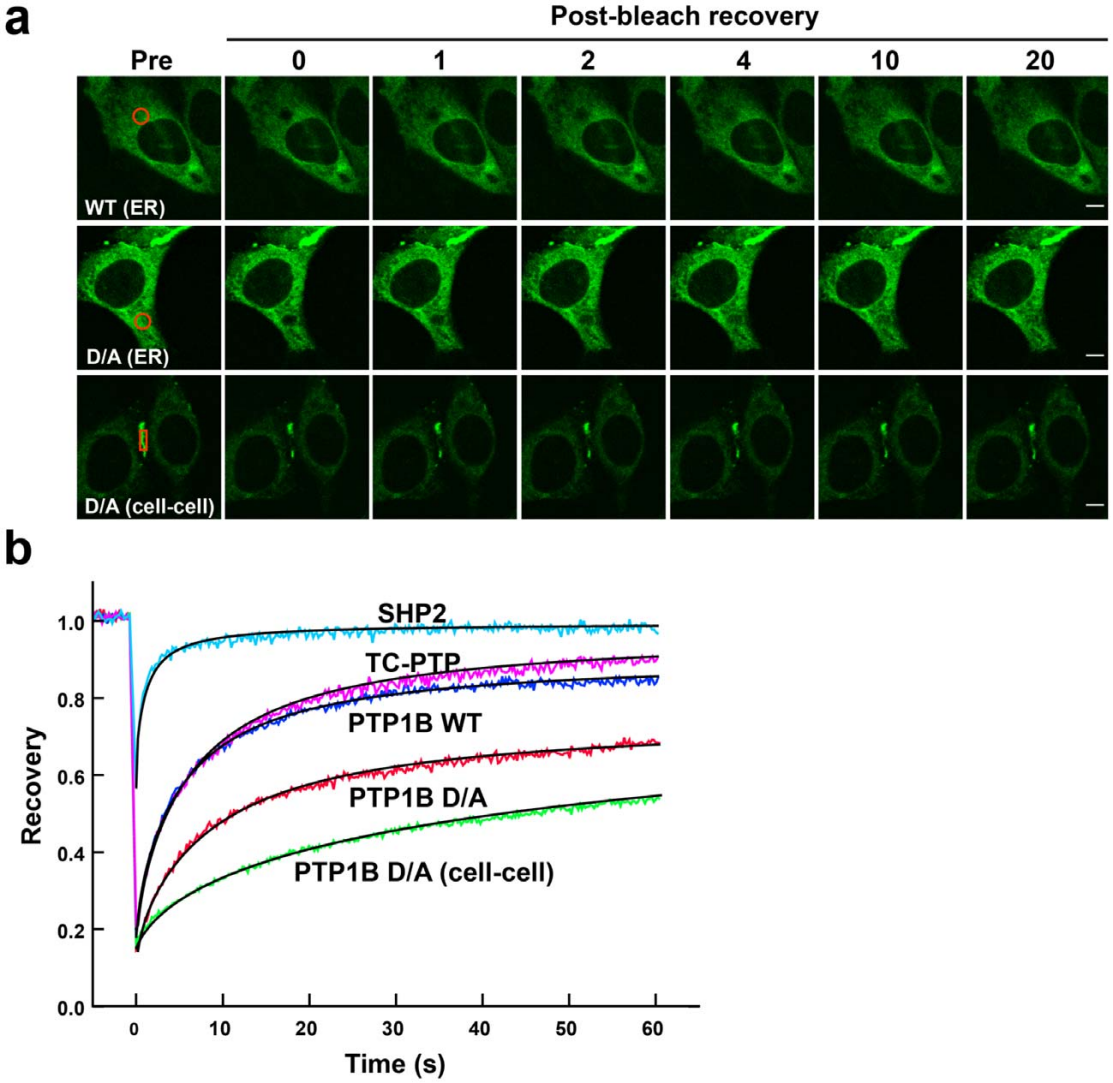
Assessment of PTP1B mobility using Fluorescence Recovery after Photobleaching. (**a**) PTP1B-null fibroblasts expressing PTP1B WT-GFP (upper panel) were photobleached in the indicated regions at the ER (circle), and fluorescence recovery was monitored for up to a minute (images are shown for the first 20 sec). Cells expressing PTP1B D/A-GFP (middle and lower panels) were photobleached either at the ER (circle) or at regions of cell-cell contact (rectangle). Scale bars correspond to 5 µm. (**b**) Quantification of intracellular fluorescence recovery in multiple cells expressing PTP1B WT, PTP1B D/A, TC PTP or SHP2 (see Fig. S2 a–d). Also shown is the fluorescence recovery of PTP1B D/A at regions of cell-cell contact (see Fig. S3e). For each PTP, actual data points are presented in color, whereas solid black lines represent fits to our mathematical model. See main text and Supplementary Materials for a detailed discussion of modeling and fitting.

The photoactivation experiments were complemented with fluorescence recovery after photobleaching (FRAP) studies [28], . GFP-labeled PTP1B WT or PTP1B D/A, expressed in randomly growing PTP1B-null cells, was photobleached irreversibly at the ER (circular area, 4 µm diameter) or at regions of cell-cell contact (rectangular area, 5 µm length/1 µm width), and the recovery of fluorescence in the photobleached area, caused by the diffusion of unbleached PTP1B, was monitored (Fig. 4a). Diffusion and binding parameters were determined from the acquired data by fitting the recoveries to an exact mathematical solution based on a simplified geometry (Fig. 4b and mathematical modeling section in Methods). For assessing intracellular diffusion, recoveries for each molecule were fit to a cylindrical model that had two free parameters: the effective diffusion constant and the immobile fraction. The effective diffusion constant measures the combined effects of actual diffusion speed (cytoplasmic or along the ER, in the absence of interactions), as well as any transient binding interactions that can slow molecular mobility. The immobile fraction reflects the fraction that does not recover (at least on the timescale of several minutes). The fitted effective cytoplasmic diffusion constant (D_eff_) of PTP1B WT was 0.23+0.01 µm^2^/s with an immobile fraction of 0.12+0.02 (mean + S.E.M.; n = 11 cells). This diffusion constant was comparable to that of the closely related, ER-anchored TCPTP (0.20+0.01 µm^2^/s) which, however, had a statistically insignificant immobile fraction (0.04+0.03; n = 12) and other ER-anchored proteins [31], [32], but was significantly lower than the predominantly cytosolic Src homology-containing tyrosine phosphatase 2 (SHP2) (1.0+0.1 µm^2^/s with a statistically insignificant immobile fraction of 0.04+0.03; n = 9). The effective diffusion of PTP1B D/A in the ER was three-fold lower than that of PTP1B WT: 0.070+0.003 µm^2^/s, with a highly significant immobile fraction of 0.28+0.02 (n = 13). This lower diffusion constant (due to a slower turnover for the transient interactions) and higher immobile fraction (indicating an increased amount of stable associations) presumably reflect interactions of PTP1B D/A with substrate(s) in the ER [15]. The slower, but still efficient recovery of PTP1B D/A - on a timescale of 10 seconds - automatically sets a lower limit on the dissociation rate for transient interactions of roughly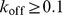 s^−1^ (a slower dissociation rate is irreconcilable with the observed efficient recovery).

The recovery of PTP1B D/A fluorescence at regions of cell-cell contact was even slower (Fig. 4a, b). The slower recovery might be explained in a number of ways, including the peripheral location of the FRAP region (replenishing proteins must on average traverse a larger distance and can approach from only one direction), the amount of PTP1B D/A that accumulates at regions of cell-cell contact (see mathematical model and Fig. S3f), and/or a slow off-rate from binding sites at regions of cell-cell contact. To help differentiate between these possibilities, we fit the recoveries to an exact mathematical solution that employed a simplified rectangular geometry. This model implicitly accounts for reversible binding of PTP1B to its substrate(s) in the ER by assuming the same effective diffusion constant determined above from the FRAP measurements for PTP1B D/A in the ER, but *explicitly* accounts for enzyme-substrate binding (both transient and immobilizing) of the accumulated fraction at the region of cell-cell contact (see mathematical model). Estimation of the exact immobile fraction at regions of cell-cell contact was hampered because we were unable to track recovery to completion due to photobleaching and cell movement (on timescales greater than one minute). Models assuming no immobile fraction or up to 30%-70% (depending on the cell) fit the data equally well (the fits shown in Figs. 4b and S3e assume no immobile fraction). This uncertainty concerning the exact immobile fraction did not, however, affect our ability to estimate any of the other parameters. Assuming an approximate accumulated amount of roughly ten percent based on the images, the model could be reduced to the determination of two parameters: the dissociation rate (of the transient interactions) and the cell size. Additionally, for most cells, only a lower limit on the dissociation rate could be determined (for all cells, the dissociation rates were consistent with 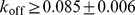 s^−1^), implying that the recoveries were limited only by the cell size (diffusion-limited recovery). The average cell size determined solely from the model fits was 28 µm, which is comparable to the observed cell sizes and demonstrates the overall self-consistency of our model with the observed properties of the cells. The roughly factor-of-two differences in recovery speeds from cell to cell can easily be attributed to the observed differences in cell size, though they could also arise from slight differences in the amount of accumulation at the cell-cell contact region. Interestingly, the required rapid turnover of transient interactions at these cell-cell contact sites (<10 seconds), and the fact that limits on the immobile fraction are compatible with the actual immobile fractions observed in intracellular regions, suggest that the affinity of PTP1B for its substrate(s) at regions of cell-cell contact is likely similar to that at other locations in the cell. The slower recovery at the cell-cell contact region can therefore be completely accounted for by the geometry of the bleaching and, in particular, the amount of accumulated PTP1B-D/A at the cell-cell contact region. The observed accumulation of PTP1B D/A is consistent with a higher concentration of PTP1B substrates at these regions (or generally on the PM) as compared with the distributed substrates found throughout the cellular interior, but is specifically not due to tighter binding of PTP1B to substrates found at regions of cell-cell contact.

### PTP1B Recruitment to Regions of Cell-cell Contact

**Figure 5:**
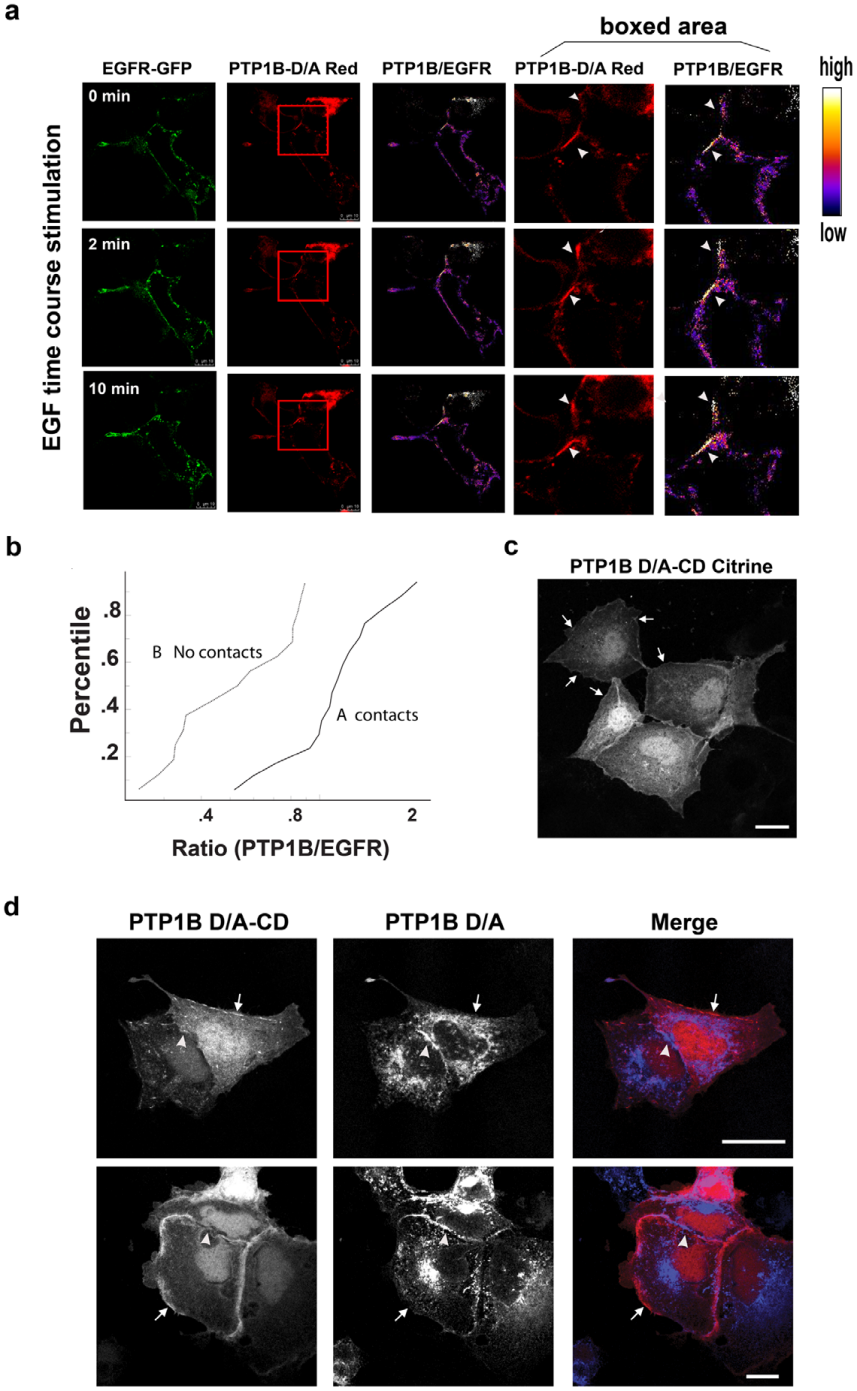
Quantification of PTP1B recruitment to regions of cell-cell contact. (**a**) Cos-7 cells co**-**expressing PTP1B D/A-dHcRed and EGFR-GFP were stimulated with EGF (100 ng/ml), and monitored by confocal time lapse microscopy. Ratio images (right panels) were generated by dividing PTP1B D/A by EGFR fluorescence following image processing as described in Materials and Methods. Scale bars correspond to 10 µm. (**b**) Distribution of PTP1B/EGFR intensity ratios in regions of cell-cell contact versus no contact (n = 21 cells). The difference between the two data sets is highly significant (p<0.001, Kolmogorov-Smirnov test). (**c**) Cells expressing mCitrine-tagged PTP1B D/A CD, shown at 5 min after EGF stimulation. Note that this mutant form of PTP1B can access substrates along the entire PM (arrows). (**d**) Cells co-expressing mCitrine-tagged PTP1B DA CD and mCherry-tagged PTP1B-D/A following EGF stimulation (5 min). Peripheral regions (regions without cell-cell contact) that show accumulation of mCitrine-tagged PTP1B D/A CD are indicated by arrows, whereas regions where ER-anchored PTP1B excluded mCitrine-tagged PTP1B D/A CD are indicated by arrowheads. Scale bars in (c) and (d) correspond to 20 µm.

We next analyzed the dynamics of PTP localization at regions of cell-cell contact in response to stimulation of the EGFR, an established PTP1B substrate (Fig. 5a). Cos-7 cells were co-transfected with PTP1B D/A-dHcRed and EGFR-GFP, and the localization of the two proteins was monitored by confocal time-lapse microscopy. Although the EGFR was expressed uniformly on the cell surface, we observed accumulation of mutant PTP1B at regions of cell-cell contacts (Fig. 5a, arrowheads) even in the absence of EGF treatment. Stimulation with EGF led to rapid recruitment of additional dHcRed-labeled PTP1B D/A to these regions (Fig. 5a, arrowheads, 2, 10 min). Importantly, the rapid EGF-induced PTP1B D/A recruitment to regions of cell-cell contact was not paralleled by increased localization of the EGFR in these regions, as ratiometric analyses showed increased PTP1B/EGFR fluorescence ratios at regions of cell-cell contact in response to EGF stimulation (Fig. 5a, white regions in PTP1B/EGFR ratio images). The increased PTP1B/EGFR ratio at the PM was observed only at regions of cell-cell contact, even though EGFR concentration, and presumably the amount of tyrosyl phosphorylated EGFR (also see below), was the same at both locations. Quantification of PTP1B D/A localization at cell-cell contacts (contacts), compared with PM regions not in contact with neighboring cells (no contacts) in multiple cells, confirmed that the ratio of PTP1B/EGFR fluorescence intensities was significantly higher at regions of cell contact compared to other areas of the cell membrane (Fig. 5b); this difference between the data sets “contacts” and “no contacts” was highly significant (p<0.001; Kolmogorov-Smirnov test).

To determine whether ER localization of PTP1B facilitates its ability to access substrates at regions of cell-cell contact, we analyzed the localization of PTP1B D/A lacking its ER-targeting domain (PTP1B D/A CD-mCitrine) in MDCK cells stimulated with EGF for 5 minutes (Fig. 5c). Unlike full length PTP1B D/A, PTP1B D/A CD-mCitrine was found in the cytosol and could interact with substrates all over the PM, with no apparent preference for substrates at regions of cell-cell contact. This result provides further evidence that the density of PTP1B substrates is similar at regions of cell-cell contact and the rest of the PM. We then co-expressed PTP1B D/A CD-mCitrine with ER-bound PTP1B D/A mCherry, and stimulated cells with EGF for 5 minutes. Consistent with our previous observations, the ER-bound form of PTP1B D/A preferentially accessed the PM at regions of cell-cell contact. On the other hand, the ER domain mutant PTP1B (free), which is localized throughout the cell and at the cell periphery, was actually displaced from regions of cell-cell contact (Fig. 5d). We interpret these findings as indicating that the high local density of ER-anchored PTP1B (which is confined to a two-dimensional surface) can outcompete the freely diffusible (soluble) catalytic domain-only mutant in regions where the ER and PM are in close contact. Collectively, these data demonstrate that ER-bound PTP1B can only reach its PM-localized substrates at regions of close ER-PM contact, and indicate that the ER anchor plays an important role in restricting PTP1B interactions with PM substrates mainly to regions of cell-cell contact.

### The ER is Polarized Towards Regions of Cell-cell Contact

**Figure 6:**
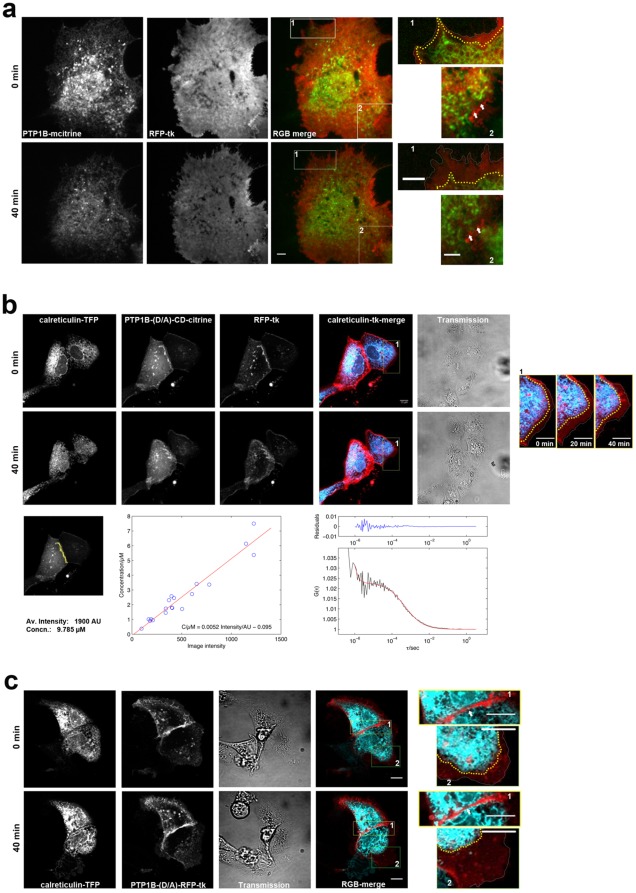
The ER is specifically polarized towards regions of cell-cell contact. (**a**) Cos-7 cells co**-**expressing PTP1B WT-mCitrine and RFP-TK were treated with nocodazole (33 µM) and imaged for 40 min using TIRF microscopy. Two regions of interest “1” and “2” are highlighted in the “Merge” images and magnified in the far right panels. “1” shows a peripheral region of the cell with no cell-cell contact. The PM outline is traced in white, the ER outline is traced in yellow, and ER retraction relative to the PM can be seen as an increase in distance between the two traced outlines after 40 min of nocodazole treatment. “2” shows a region of cell-cell contact (indicated by arrows). Nocodazole treatment leads to ER retraction from peripheral regions but not from areas of cell-cell contact. (**b**) MDCK cells expressing calreticulin-TFP, PTP1B D/A CD-mCitrine, and RFP-TK. On the right hand side, a magnified region of interest (rectangle) shows peripheral regions of the ER. Lower left panel shows calculated concentration of PTP1B D/A CD in a cell-cell contact region of interest based on fluorescence calibrated imaging [38]. Lower central panel: correlation of the average image intensity measured in the cytoplasm of cells expressing mCitrine-PTP1B D/A CD to the absolute concentration, as determined from FCS measurements. A linear fit (red line) yielded the calibration function used to calculate absolute concentrations from image intensities. Lower right panel: Example of an auto-correlation G(τ) curve of PTP1B D/A CD-mCitrine (black line) fit by a model accounting for anomalous diffusion and a triplet term (red line). (**c**) MDCK cells co-expressing calreticulin-TFP and PTP1B D/A-RFP-TK were treated with nocodazole and imaged by confocal microscopy for 40 min. “1” shows regions of cell-cell contact; note how the ER does not retract from these points after nocodazole treatment. “2” shows a peripheral region of the PM; note the distance increase between the ER and PM after nocodazole treatment. All scale bars correspond to 10 µm.

The above findings indicate that the ER is specifically organized and oriented towards regions of cell-cell contact, which raised the possibility that PTP1B might play a role in this process. Microtubules contribute to the formation and stabilization of the ER network [33], [34]. To destabilize the ER network, we disrupted microtubules using nocodazole, and ER retraction at the periphery and regions of cell-cell contact was monitored using total internal reflection microscopy (TIRF) [35]. Cos-7 cells were co-transfected with PTP1B WT-mCitrine and RFP-tagged TK-Ras (a general PM marker to identify areas of cell-cell contact and account for cell shape changes) [36]. Consistent with previous reports [37], we observed ER collapse, specifically the loss of peripheral tubular ER through its conversion into sheet-like structures that retracted from the cell periphery, after nocodazole treatment (Fig. 6a and Fig. S4a). The extent of ER reorganization varied between experiments depending on cell confluence, cell type and shape. Nevertheless, the ER always retracted from peripheral regions but seemed to persist at regions of cell-cell contact. In isolated cells expressing PTP1B D/A, the ER retracted only partially after nocodazole treatment, with long stretches of tubular ER still attached to the PM at sites of focal adhesion to the glass coverslip (Fig. S4b).

These observations raised the possibility that, rather than (or in addition to) the ER directing PTP1B towards substrates at points of cell-cell contact, PTP1B interactions with substrates might help polarize the ER towards these regions. We reasoned that if this was the case, then outcompeting endogenous PTP1B for substrate interactions by overexpressing PTP1B D/A CD should allow the ER to retract from regions of cell-cell contact after nocodazole treatment. PTP1B WT localization is not altered by PTP1B D/A co-expression (Fig. S5). PTP1B D/A CD-mCitrine, monomeric Teal fluorescent protein (mTFP)-tagged Calreticulin (ER marker), RFP-tagged TK-Ras and EGFR were co-transfected into MDCK and Cos-7 cells. After starvation, cells were stimulated with EGF (100 ng/ml) for 5 minutes (to increase phosphorylation and recruitment of PTP1B D/A CD to its substrates), and then cells were treated with nocodazole. The ER retracted from peripheral regions as observed before, but failed to retract from regions of cell-cell contact (Fig. 6b). To assess whether the expression of PTP1B D/A CD was high enough to compete for substrates with the endogenous pool of PTP1B, we measured the absolute concentration of ectopically expressed PTP1B D/A CD across the cell by correlating the image intensity of cytoplasmic PTP1B D/A CD to its concentration, as determined by fluorescence correlation spectroscopy [38]. A linear fit to this correlation enabled the estimation (by extrapolation) of PTP1B D/A CD concentrations at any point in the cell. The maximal concentration of PTP1B D/A CD was observed at regions of cell-cell contact and was approximately 9 µM (Fig. 6b). In the immediate cytoplasmic vicinity of these cell-cell contacts, the concentration of PTP1B D/A CD remained in the µM regime, which is comparable to the K_D_ for PTP1B D/A-substrate interactions [17]. We therefore conclude that over-expressed PTP1B D/A CD should efficiently compete with endogenous PTP1B for substrates at the PM of cell-cell contacts. However, endogenous PTP1B may have an additional advantage, as it is anchored to the 2D ER, which may give it a geometrical advantage in its search for substrates (higher k_on_) over the cytosolic PTP1B D/A CD (invalidating arguments based on the K_D_, which assumes a search in 3D). To even overcome the high-local concentration of ER-bound PTP1B, we performed competition experiments with PTP1B D/A lacking the ER-targeting domain but fused to the C-terminal membrane anchoring residues of K(B)Ras (TK-Ras) (hereafter referred to as PTP1B D/A-RFP-TK). We reasoned that anchoring PTP1B D/A to the PM should give it an enhanced geometric, and consequently kinetic, advantage in substrate interaction over the endogenous ER-bound enzyme. We then co-expressed PTP1B D/A-RFP-TK with TFP-tagged calreticulin and unlabeled EGFR, and treated Cos-7 cells with nocodazole as described before. Again, the ER did not retract from regions of cell-cell contact compared with other regions of the PM (Fig. 6c). Taken together, these findings clearly indicate that PTP1B-substrate interactions do not by themselves stabilize the polarization of the ER to regions of cell-cell contact. Rather, intrinsic polarization of the ER towards points of cell-cell contact likely directs PTP1B towards these regions.

### Regulation of Signaling at Regions of Cell-cell Contact by PTP1B

**Figure 7:**
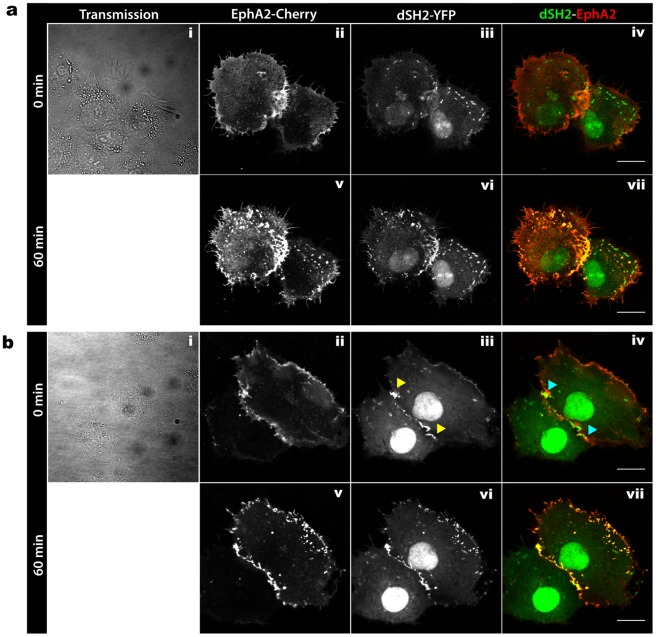
PTP1B accesses specific substrates at cell-cell contacts. (**a, b**) Cos-7 cells co**-**expressing EphA2-mCherry (panels ii, v) and dSH2-YFP (panels iii, vi) were incubated for 1 hour at 37°C with PTP1B allosteric inhibitor (539741 PTP1B inhibitor, 250 µM), and monitored by confocal time lapse microscopy. Transmission images (panel i) show the cell confluency and help to define regions of cell-cell contact. Intensity merge images (panel iv, vii) showing the spatial localization of EphA2 (red) an dSH2 (green) before (iv) and after (vii) PTP1B inhibitor treatment. Note the markedly increased co-localization (orange) at regions of cell-cell contact after inhibitor treatment, indicating increased EphA2 phosphorylation specifically in this region. Scale bars correspond to 20 µm.

The above findings indicated that there is an orientation of the ER towards regions of cell-cell contact, which raised the possibility that PTP1B may play a significant role in the regulation of signaling at these cites. To test this hypothesis, we determined the effects of PTP1B inhibition on the phosphorylation of substrates found at regions of cell-cell contact. Cos-7 cells were transfected with mCherry-tagged EphA2, a RTK that is closely related to EphA3 (a recently described substrate of PTP1B) [39]. Tyrosine phosphorylation was monitored using the co-transfected YFP fused to SH2 domain of pp60^src^ (dSH2-YFP) [40]. Cells were treated with an allosteric PTP1B inhibitor (539741) [41], which exhibits higher selectivity compared with active site inhibitors, and then monitored by confocal time-lapse microscopy for 60 minutes. Before inhibitor treatment, EphA2 showed typical membrane distribution of a RTK, with occasional accumulation at regions of cell-cell contact (Fig. 7a, b, panel ii). In addition, dSH2-YFP showed expression in the nucleus, punctuate clusters surrounding the cell typical of focal adhesions (Fig. 7a, panel iii), and in some cases at regions of cell-cell contact (Fig. 7b, panel iii yellow arrowheads). Minimal spatial overlap was observed between dSH2 and EphA2 prior to inhibitor treatment, even at regions of cell-cell contact (Fig. 7a, b panel iv cyan arrowheads). On the other hand, treatment with PTP1B inhibitor for 1 hr led to extensive increase in spatial overlap between dSH2 and EphA2 especially at regions of cell-cell contact (Fig. 7a, b, panel vii). The increased tyrosine phosphorylation of EphA2 upon PTP1B inhibition suggests that EphA2 is a potential PTP1B substrate at these sites. These findings reveal that the ER-bound PTP1B, by virtue of the organization and polarization of ER towards points of cell-cell contact, can access its potential substrates and regulate their activity at these regions.

## Discussion

One of the earliest discoveries about PTP1B was its localization to the cytosolic surface of the ER [12], [13]. This result was initially quite surprising, because biochemical and genetic studies soon established that PTP1B dephosphorylates several RTKs, including the PDGF, EGF and insulin receptors [2], [3], [42], [43], [44]. We subsequently provided an answer to this topological dilemma with the finding that RTKs encounter PTP1B only after they are endocytosed [14], a finding confirmed and extended by several other groups [15], [16], [45]. Nevertheless, other work indicates that PTP1B can access PM substrates at points of cell-cell contact [21]. These results raise an apparent paradox: why can PTP1B dephosphorylate some substrates only after endocytosis, whereas others can be targeted while at the PM? The results herein provide a solution to this paradox and present a novel mechanism of how ER-PM signaling at regions of cell-cell contact is regulated. This regulation is important in that it is observed in different cell types, including those that do and do not form tight junctions. Our findings clearly demonstrate that interactions between the PM and the ER are not ubiquitous, but rather are restricted to regions of cell-cell contact. In these regions, the ER network appears to be specifically organized and oriented such that it is juxtaposed to the PM. An analogous mechanism might also account for PTP1B interactions with substrates at cell-matrix contacts.

If we envision that the ER can reach the PM everywhere in a stochastic, dynamic way, then the local concentration of substrates should determine the amount of PTP1B D/A at regions of cell-cell contact. Conceivably, substrate concentrations at regions of cell-cell contact are higher than elsewhere in the PM, in which case a critical amount of substrate(s) must be present to enable the ER-bound phosphatase to access them while they are still localized to the PM. Our photoactivation and FRAP studies show that PTP1B D/A rapidly moves in and out of regions of cell-cell contact (replenished by the ER pool), with a residence time of <10 s (Figs. 3, 4). These findings are consistent with the presence of a large number of PTP1B binding sites, presumably representing substrates in these regions. Nevertheless, when the EGFR is at the same concentration in the PM and regions of cell-cell contact, interaction (without prior endocytosis) occurs only at the latter location (Fig. 5). Furthermore, expression of the soluble cytosolic domain of PTP1B D/A does not result in a specific enrichment of this protein to points of cell-cell contact after EGF stimulation; rather, this protein labels the PM uniformly, thereby excluding the possibility that substrate density alone dictates where PTP1B interacts with substrates at the PM. Varying rates of endocytosis (at cell-cell contacts versus other areas of the PM) also might account for the differential ability of PTP1B to access substrates at the PM. Arguing against this model, though, we previously blocked RTK endocytosis using dominant negative dynamin, but were unable to detect direct interaction between PTP1B and PM-bound RTKs using fluorescence lifetime imaging microscopy (FLIM) [14]. Taken together, these findings strongly suggest that additional factors are instrumental in enabling ER-bound PTP1B to access substrates specifically at regions of cell-cell contact. These factors are likely responsible for polarizing the ER towards regions of cell-cell contact, thereby creating specialized zones of ER-PM interaction at cell-cell contacts in which PTP1B (and conceivably, other ER proteins) may access the PM.

There are precedents for the existence of specific ER-PM signaling compartments. Studies using *S. cerevesiae* indicate that membrane curvature may help to define functionally distinct sub-domains in the ER [46], [47], [48]. Whether an analogous mechanism creates ER sub-domains near regions of cell-cell contact in mammalian cells remains to be determined. In skeletal muscle, the sarcoplasmic reticulum junctophilin proteins bind to PM components to form junctional contacts [49]. A new paradigm for ER-PM signaling has been proposed for the ER calcium sensor; stromal interaction molecule 1 (STIM 1) [50]. After ER calcium depletion, STIM 1 oligomerizes, and due to enhanced avidity, binds to and activates store-operated calcium channels at the PM [50], [51], [52]. Ultrastructural studies reveal that upon calcium depletion, STIM 1 induces the formation of cortical ER (cER), which is composed of large sheets that are closely opposed to the PM but still connected to conventional ER cisternae [53]. The distance between cER and PM (on average 8.3 nm in Epon sections) is comparable to the distance between ER-bound PTP1B and the PM in our EM studies (Fig. 2). Moreover, mammalian STIM proteins have a lysine-rich sequence that is similar to the yeast peripheral protein Ist2 (a transmembrane protein that is localized to cER in yeast). Remarkably, Ist2 enrichment at the cER of mammalian cells directly modulates the formation and maintenance of this ER subregion, and cER induction *in vivo* is dependent on microtubules and the coat protein complex I (COPI) [54]. It will be important to determine whether cER or some other higher order ER structure promotes PTP1B interactions with substrates at cell-cell contacts. The identity of these substrates and their contribution to cell-cell signaling requires further investigation. Recent studies identified interactions between PTP1B and EphA3 and revealed that the interaction can occur at the plasma membrane at areas of EphA3/ephrin-mediated cell-cell contact [39]. In the current study, we report increased EphA2 tyrosine phosphorylation at regions of cell-cell contact upon PTP1B inhibition, suggesting that EphA2 is putative substrate of PTP1B at these sites. Other proteins known to reside at cell-cell contacts, such as p120 catenin and Zonula Occludens (ZO-1), are hyper-phosphorylated in PTP1B-null fibroblasts, and represent additional candidate PTP1B substrates in these regions [55]. Collectively, our studies suggest that the ER plays a dynamic role in regulating signaling at regions of cell-cell contact via PTP1B and highlight ER-PM interactions as an emerging new paradigm in cellular signaling.

## Materials and Methods

### Cell Culture, Antibodies and Reagents

PTP1B-null fibroblasts (Haj et al. 2003), MDCK and Cos-7 cells (both obtained from ATCC) were cultured on 35 mm glass bottom culture dishes (MatTek Corporation) or 4-well Lab-Tek chambers (Nunc) in Dulbecco’s modified Eagle medium (DMEM) with 10% fetal calf serum (FCS). Unless otherwise indicated, cells were transfected using Lipofectamine 2000 (Invitrogen), as described by the manufacturer, and incubated at 37°C and 5% CO_2_ for at least 12–18 hours before imaging. For live cell experiments, the culture medium was replaced with CO_2_-independent imaging medium. Antibodies against mouse β-catenin (#610153) were purchased from BD Biosciences, fluorescein-conjugated secondary antibodies were from Jackson Immunoresearch, and nocodazole was purchased from Sigma. PTP1B allosteric inhibitor 3-(3,5-Dibromo-4-hydroxy-benzoyl)-2-ethyl-benzofuran-6-sulfonicacid-(4-(thiazol-2-ylsulfamyl)-phenyl)-amide (Cat #539741) was purchased from Calbiochem.

### Plasmid Constructs

PM-anchored PTP1B D/A was generated by inserting cDNA encoding the catalytic domain of human PTP1B (residues 1–407) flanked by Age I restriction sites into pRFP-tkB-C1 vector (gift of Dr. M. Lackmann). pRFP-tkB-C1 (hereafter referred to as RFP-TK) consists of the C-terminus membrane targeting domain and part of linker domain of K(B)Ras downstream of mRFP.

### Immuno-electron Microscopy

Cells were fixed with 4% paraformaldehyde in 0.2 M sodium phosphate buffer, pH 7.4 for 5 min, followed by 2% paraformaldehyde in 0.1M sodium phosphate buffer for 30 min, then rinsed with PBS and quenched with 50 mM glycine in PBS for 10 min. After adding 2 ml of 1% gelatin, the cells were collected by scraping the monolayer, centrifuged, resuspended in 10% gelatin [56], and then centrifuged again for 1 min. After cooling on ice for 30 min, the pellet was cut into small cubes, which were infiltrated with 2.3 M sucrose overnight, mounted on pins and frozen in liquid nitrogen. Cryosections were prepared using an ultra-cryomicrotome (Leica) and collected with a 1∶1 solution of 2.3 M sucrose and 2% methyl cellulose [57]. Immunogold labeling with anti-PTP1B (FG6) antibodies (diluted 1/10–1/50) was performed and revealed with protein-A gold conjugate (Utrecht University). Cryosections were viewed at 100kV on a Biotwin EM scope (FEI) equipped with a SIS Keen View 1.3×1 K CCD camera.

For cryo-electron microscopy of vitreous sections (CEMOVIS), samples were treated as previously described [58]. Briefly, cells were grown on ACLAR film (Science Services, Eppelheim, Germany), fixed using HPM100 high pressure freezer (Leica Microsystems, Germany) and cut at –150°C. Samples were analyzed using a JEM-1400 electron microscope (JEOL Germany) at 120 kV equipped with F-416 CCD camera (TVIPS, Gauting, Germany). For vitreous sections, a cryo-holder model 626 (Gatan, Pleasanton, CA) was used.

### Confocal Imaging, Photoactivation and Time Lapse Microscopy

Confocal imaging and photoactivation were performed using an Olympus FlowView FV 1000 microscope with a 63×/1.4 N.A oil objective. Experiments were performed in an environmental box in which live cells were maintained at 37°C and 5% CO_2_. Photoactivation was performed in the region of interest using a 413 nm laser in the region of interest, and continued imaging of the photoactivated pool was performed using a 488 nm laser. All studies were conducted on randomly growing cells.

For confocal time-lapse microscopy, Cos-7 cells were transiently co-transfected with EGFR-GFP and PTP1B-dHcRed using Fugene 6 (Roche Biochemicals). Cells were serum-starved, stimulated with EGF (100 ng/ml) and then subjected to confocal time-lapse imaging. Images were captured using a confocal laser microscope (Leica TCS-SP5) with a 63×/1.4 N.A oil objective at 12-bit resolution. Image processing and quantitative analysis were performed using Image J. For ratiometric analysis, images were converted to 32-bit (floating point) format and thresholded. Background was defined as not a number (nan) and ratio images were then generated by dividing PTP1B D/A dHcRed by EGFR-GFP images.

### Fluorescence Recovery after Photobleaching

Photobleaching was performed on the temperature-controlled stage of a Leica SP2 AOBS Sirius microscope equipped with a 63×/1.4 N.A oil immersion lens. GFP was excited using a 488 nm Argon laser, and fluorescence was monitored at 0.203 sec intervals. Fifty pre-bleach images were recorded with 4% laser power of the 488 line every 0.203 sec. Consequently, a region of interest (outlined in the figure) was photobleached using 100% laser power of the 456, 476, 488 and 496 lines. Recovery of fluorescence was monitored over the course of 300 whole-cell scans (with a scan interval of 0.203 sec).

For bleaching of PTP1B WT-GFP and PTP1B D/A-GFP in the ER, a circular region of 4 µm diameter was used, whereas for bleaching at cell-cell contacts, we used a rectangular area of 5 µm length and 1 µm width. Cells with varying levels of transfection ranging from high to low PTP1B WT and D/A expression were studied. Mean fluorescence intensities in the FRAP region and for the whole cell (including the FRAP region) were recorded, and the background was subtracted. The FRAP region and whole cell profiles were then individually normalized to their pre-bleach values (obtained by averaging the 11 images immediately preceding bleaching), and the final FRAP recovery profile was obtained by dividing the normalized FRAP region intensity by the normalized whole cell intensity. This final step removed the global fluorescence decrease due to photobleaching during initial bleaching (∼5% for FRAP experiments in the ER and 5–20% for FRAP experiments in the cell-cell contacts), as well as any gradual bleaching occurring during acquisition of the recovery (less than a few percent for all experiments). Mathematical modeling was used to fit the recoveries (see Supplementary Materials).

### Total Internal Reflection Fluorescence Microscopy

Cos-7 cells were transiently co-transfected with RFP-TK and PTP1B WT-mCitrine or PTP1B D/A-mCitrine using Effectene (Qiagen). Cells were plated onto glass-bottom 35 mm dishes (MatTek) and then were incubated in CO_2_-independent medium supplemented with 10% FCS and 2.5 mM L-glutamine. Images were acquired with an inverted Olympus Cell̂R microscope configured for triple line total internal reflection fluorescence, using a 60× PLAPO/TIRFM-SP oil immersion objective, with 1.45 N.A at 16-bit resolution in each channel.

### Fluorescence Calibrated Confocal Time-lapse Microscopy

Fluorescence-calibrated time lapse imaging was performed on a Zeiss LSM 510 Meta confocal microscope equipped with a ConfoCor 3 unit and a C-Apochromat 40×/1.2 N.A water immersion objective. Experiments were performed in an environmental box in which the sample and objectives were maintained at 37°C. mTFP and mCitrine were excited with 458 nm and 514 nm argon lasers, respectively, whereas RFP was excited with a 561 DPSS laser. The fluorescent light was passed through a NFT 565 beam splitter and detected with PMT detectors through a BP 475–525 band pass filter for mTFP, a LP 530 long pass filter for mCitrine, and a LP 575 long pass filter for RFP. To minimize cross-talk between individual channels, mTFP and RFP were recorded simultaneously, whereas mCitrine was imaged separately. Pinhole diameter was set to 192 µm for mTFP, 296 µm for RFP, and 1000 µm for mCitrine. Images were recorded at a resolution of 512×512 pixels (0.15 µm/pixel) and a bit depth of 12 bit. For time-lapse imaging, single-plane multi-color images were recorded at 1 minute intervals.

To correlate the image intensity of mCitrine to absolute protein concentrations, we acquired single-channel images of cells expressing mCitrine-PTP1B D/A CD at exactly the same settings as for the time-lapse experiments. Fluorescence correlation spectroscopy (FCS) measurements were performed at randomly chosen positions in the cytoplasm of the same cells. Background-corrected average image intensities were determined in a 1 µm radius around the FCS measurement point. For FCS, mCitrine was excited with the 514 nm line of an argon laser, while an APD detector recorded the fluorescence through a LP 530 long pass filter and a 70 µm pinhole. The raw intensity data were correlated using the microscope manufacturer’s software, and for each measurement, correlation curves were averaged for ten consecutive 10-second intensity recordings. Averaged correlation curves were fit by using a model accounting for anomalous diffusion and a triplet term in order to determine the average number of particles in the confocal observation volume, using a non-linear least squares minimization algorithm implemented in Matlab (MathWorks). Confocal volume was determined from calibration measurements of an Alexa 546 dye solution, assuming a diffusion coefficient of 280 µm^2^/s at 25°C that was extrapolated to 308 µm^2^/s at 37°C.

### Mathematical Model for Fitting FRAP Data

For the circular FRAP in the ER (PTP1B WT and PTP1B D/A), we used the analytic formula for a cylindrical region in an infinite medium [59]:
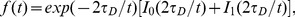
(1)where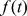 denotes the FRAP recovery (recovering from 0 to 1), I_0_(*x*) and I_1_(*x*) are the standard modified Bessel functions, and 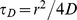 is the diffusion timescale with *r* the radius of the FRAP region and *D* the diffusion constant. Considering the case of imperfect bleaching and the possibility of an incomplete recovery due to an immobile fraction leads to the following formula:
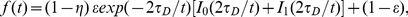
(2)where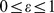 is the bleaching efficiency (with 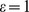 corresponding to complete bleaching) and 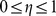 is the immobile fraction. The initial FRAP bleaching in the region of interest in our cells removed less than a few percent of the total cellular fluorescence. This justifies the use of the “infinite cell” approximation implicit in Eq. 2.

We obtained the following values for diffusion constants and immobile fractions for the four intracellular/ER FRAP data sets (taking 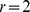 µm for the radius of the FRAP region):

D (µm2 s^−1^) 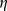 n

PTP1B WT-GFP 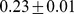 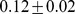 11

PTP1B D/A-GFP 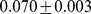 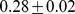 13

TCPTP-GFP 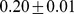 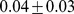 12

SHP2-GFP 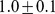 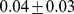 9

Each FRAP profile was fit independently, and the above values quoted are the mean and standard error of mean (S.E.M) for the indicated number of cells, *n* (individual cell profiles shown in Fig. S2). The mean and S.E.M of the diffusion constant were based on the statistics of the logarithm of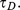 The above parameters also fit well the mean cell profile obtained by simply averaging all of the single cell recovery profiles (see Fig. 4b in the main text). PTP1B D/A, therefore, has a three-fold lower diffusion constant and a two-fold greater immobile fraction than PTP1B WT, consistent with the “trapping” nature of its catalytic region mutation. The diffusion of PTP1B WT is similar to that of another ER-bound phosphatase, TCPTP; however, the immobile fraction of TCPTP is insignificant (consistent with the few percent removal of fluorescence from the initial FRAP bleaching). All of the ER-bound proteins diffuse much more slowly than the cytosolic SHP2 (which also has an insignificant immobile fraction).

For the FRAP experiments at the cell-cell interface, we employed a Laplace transform approach similar to that used previously [60], [61]. In the latter, a 2D cylindrically-symmetric geometry was explored. Here, we have a 1D system with a non-trivial boundary condition (due to the location of the binding sites there). Because this important case was not examined before and, in particular, because the inclusion of boundary conditions is subtle, we provide a full derivation for the sake of clarity and completeness. The following equations describe the concentration of a freely diffusing fluorescent protein, 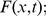 free binding sites, 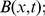 and binding sites bound with the fluorescent protein, 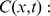


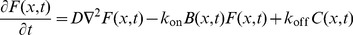
(3)

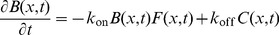
(4)

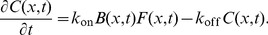
(5)


Because the binding sites are localized to the cell-cell interface, 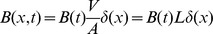 and, similarly,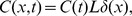 where 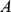 is area of the cell-cell interface (at 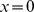) and 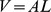 is the cell volume. This gives:
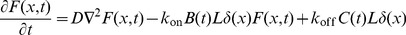
(6)

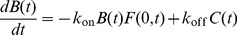
(7)

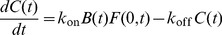
(8)


At steady state, the concentration of free binding sites, 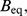 is unaffected by the FRAP process. We therefore need consider only the time dependence of 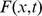 and 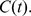 Following bleaching, the free and bound fluorescent protein concentrations split into bright and dark fractions:
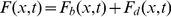
(9)

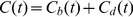
(10)


Upon substituting back into the differential equations above, these equations can be dissected into separate sets of equations (of the same form) for both the bright and dark fractions. Of course, we are only able to monitor the bright fraction:
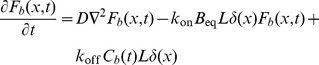
(11)

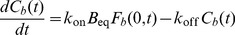
(12)


These equations are best solved by the Laplace transform, which gives:
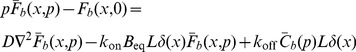
(13)

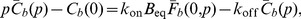
(14)where the bar indicates the Laplace-transformed function dependent on the Laplace transform variable *p*. Rearranging,
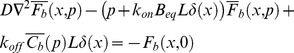
(15)




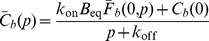
(16)


The initial concentration of the free protein is just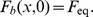 We assume an initial bleaching of the bound protein, giving
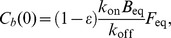
 where 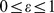 is the bleaching efficiency, with 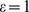 denoting complete bleaching. Substituting 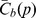 into the differential equation for 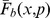 now gives:
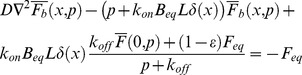
(17)


For 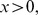


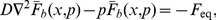
(18)with general solution:



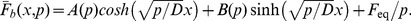
(19)The boundary condition at 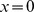 (using Gauss’s law) is
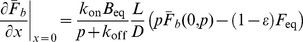
(20)and at 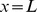 is simply 0 (hard wall, Neumann boundary condition). Using the boundary conditions to solve for the coefficients of the general solution yields:



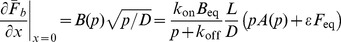
(21)

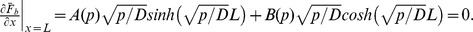


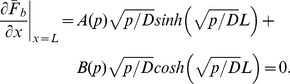
(22)


These give the following for the specific solution (at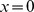):
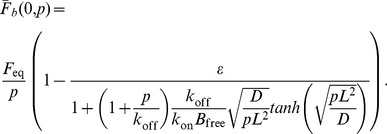
(23)


This can then be substituted into the equation for 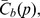 yielding
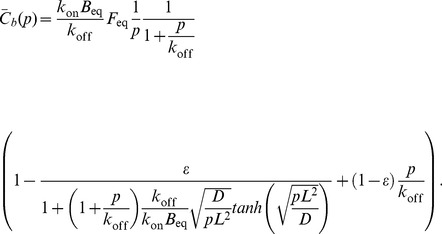
(24)


Normalizing to the pre-bleach amount of bound protein and rearranging gives the following dimensionless form for the Laplace transform of the recovery of fluorescence at the cell-cell interface:
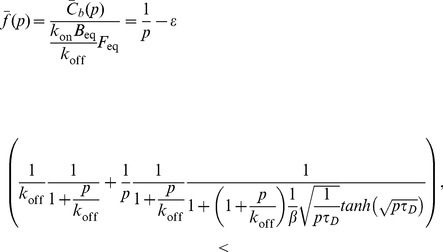
(25)where 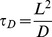 is the diffusion timescale and 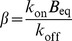 is the contrast, which is equal to the ratio of the total protein bound at the interface 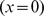 to the total free protein in the cell. The full recovery is then:



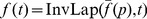
(26)

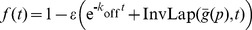
(27)with



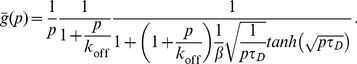
(28)From the fitting, we find that the “tanh” term is effectively 1 in our case, implying:
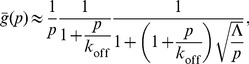
(29)with the recovery effectively depending on only two unknown parameters: 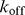 and 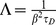 (the contrast and diffusion timescale are degenerate). But this is just the recovery one gets assuming an “infinite” cell. Here, the general solution is just:



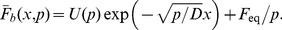
(30)The boundary condition at 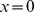 (using Gauss’s law) is
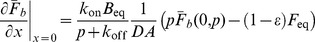
(31)

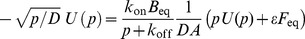
(32)


giving:



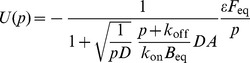
(33)

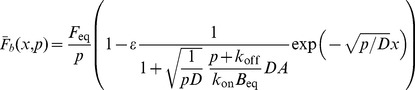
(34)

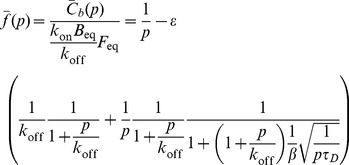
(35)


And, again,
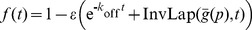
(36)now with the exact equality:



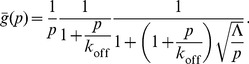
(37)The recovery (normalized only to the pre-bleach fluorescence in the FRAP region) asymptotically approaches the following value:
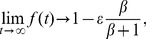
(38)which for complete initial bleaching of the bound protein 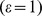 is just 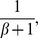 or the ratio of free protein to total protein. However, for our data analysis, we additionally normalized to the total cellular fluorescence at each time point:
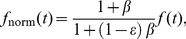
(39)which removes the reduction in intensity caused by the bleaching of the total cellular fluorescence. The bleaching efficiency is directly observed, and the diffusion constant can be obtained by FRAP in the interior of the cell (hence, our FRAP experiments with PTP1B D/A in the ER, which yielded 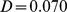 µm2 s−1). Assuming that the confocal plane we observe is representative, we find that 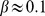 for most cells (this value, true for the entire stripe, can also be used for our experiments wherein only one third of the interface was bleached). This implies that the additional normalization in 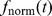 is only a few percent. It is therefore safe to ignore this (the “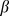 is small” limit where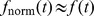) and simply fit to
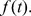


Assuming zero immobile fraction (see below), we have independently fit the recovery curve for each of the 12 cells (Fig. S2), obtaining mean and S.E.M values (based on the logarithmic values) for the two remaining free parameters 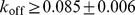 s^−1^ (residence half-life of 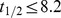 s) and 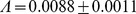 s^−1^. For most of the recovery curves, 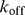 needed only to be greater than some minimal value in order to fit the data, hence the lower limit. The mean values again fit well the mean profile resulting from averaging the recovery profiles of all of the cells, as shown in Fig. 4b. Using 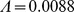 s^−1^, 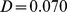 µm^2^ s^−1^, and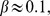 gives an effective cell length of 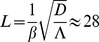 µm, which is comparable to the observed cell sizes. The FRAP recovery of PTP1B D/A at the cell-cell interface is therefore consistent with rapid turnover (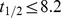 s), the ER-determined diffusion constant, the observed level of PTP1B D/A accumulation at the interface, and the cell size. From the raw images themselves (Fig. 4a), it is already clear that the recovery we observe is not an artifact, but represents a real turnover of bound protein at the cell-cell interface. As mentioned above, an immobile fraction at the cell-cell interface is not required in order to fit the recovery (in all of our displayed fits, we assume no immobile fraction). Recoveries assuming an immobile fraction also fit the data (not shown), and on a cell-by-cell basis these fits provide a useful upper limit to the immobile fraction at the cell-cell interface ranging from <30% to <70%. Theoretically, a longer acquisition time would help eliminate most of the parameter degeneracy and place a tighter limit on the immobile fraction, but observation of the actual long-term recovery is severely complicated by cell movement and morphological changes occurring on minute timescales.

All of the analysis was performed in Mathematica (Mathematica, Inc.), where we have used the inverse Laplace transform algorithms described in: http://library.wolfram.com/infocenter/MathSource/4738/and http://library.wolfram.com/infocenter/MathSource/5026/.

## Supporting Information

Figure S1 **PTP1B D/A on the surface of the ER accesses PM substrates at points of cell-cell contact.** (**a**) Serial images were acquired at successive focal planes of PTP1B-null fibroblasts expressing PTP1B D/A-GFP (Z step: 0.23 µm). Regions of cell-cell contact are indicated with arrows and magnified in the inset. Note the “honeycomb” appearance characteristic of the ER. (**b**) Cells expressing PTP1B WT or D/A were treated with the general tyrosine phosphatase inhibitor pervanadate to disrupt PTP-substrate interactions. Note the disappearance of PTP1B D/A localization at points of cell-cell contact after pervanadate treatment, indicating interaction of PTP1B D/A with PM bound substrates. Areas of cell-cell contact are indicated with an arrow. Scale bars correspond to 5 µm.
(TIF)

Figure S2 **The ER also lies in proximity to the PM in HeLa cells**. Epon section routine EM and high-resolution cryo-electron microscopy of vitreous sections (CEMOVIS) of wild type HeLa cells showing close association of ER and PM membranes (arrows). ER: endoplasmic reticulum; PM: plasma membranes of neighboring cells; asterisks: tight junctions; scale bar 100 nm.
(TIF)

Figure S3
**Quantitative assessment of fluorescence recovery in cells expressing various PTPs.** Assessment of fluorescence recovery over time of various PTPs at the ER (**a-d**) or PTP1B D/A at regions of cell-cell contact (**e**) in different cells. The fitted model curves (red) are overlaid on the experimental data (black). (**f**) FRAP recovery rates of PTP1B D/A at regions of cell-cell contact in cells with different levels of PTP1B D/A accumulation. The curves correspond to different ratios of total bound to total free PTP1B D/A in the cell, which is designated as 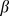 in the model (curve labels specify the values for 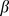 that were used). The central curve 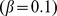 is identical to the averaged PTP1B D/A cell-cell recovery model shown in Fig. 4b. A larger accumulation at regions of cell-cell contact clearly leads to a slower recovery. Because the level of accumulation and cell length are degenerate in the model, the exact same curves would also result by varying the cell length by similar factors.(TIF)

Figure S4 **Polarity of the ER.** (**a**) Cos-7 cells co**-**expressing Calreticulin-TFP and RFP-TK were treated with nocodazole (33 µM) and imaged for 30 min using TIRF microscopy. Two regions of interest “1” and “2” are highlighted and magnified in the “Merge” images. “1” shows a peripheral region of PM with no cell-cell contact; note how the ER extends out to the peripheral PM before treatment. “2” shows a region of cell-cell contact (indicated by yellow arrow); note that the ER does not retract from points of cell-cell contact after treatment. (**b**) Cos-7 cells co-expressing PTP1B D/A and RFP-TK were treated with nocodazole and imaged for 40 min using TIRF microscopy. In the lower right panel, arrows indicate the long stretches of ER network that remain attached to sites of adhesion to the glass coverslip following nocodazole treatment; this is observed only for cells overexpressing PTP1B D/A. Scale bars correspond to 10 µm.
(TIF)

Figure S5 **PTP1B WT localization is not altered by PTP1B D/A co-expression.** Randomly growing MDCK cells were transiently co-transfected with PTP1B WT-mCitrine and PTP1B D/A-mCherry. PTP1B WT and PTP1B D/A colocalized across the entire ER; however, the D/A mutant was clearly much more highly accumulated to cell-cell contact sites. Right panel represents phase contrast images of the cells and arrows indicate sites of cell-cell contact.
(TIF)
