# Supplementary figures and images for: Regulation of Signaling at Regions of Cell-Cell Contact by Endoplasmic Reticulum-Bound Protein-Tyrosine Phosphatase 1B
Source: PLoS One. 2012 May 24;7(5):e36633. doi: 10.1371/journal.pone.0036633 (PMC3360045; doi:10.1371/journal.pone.0036633)

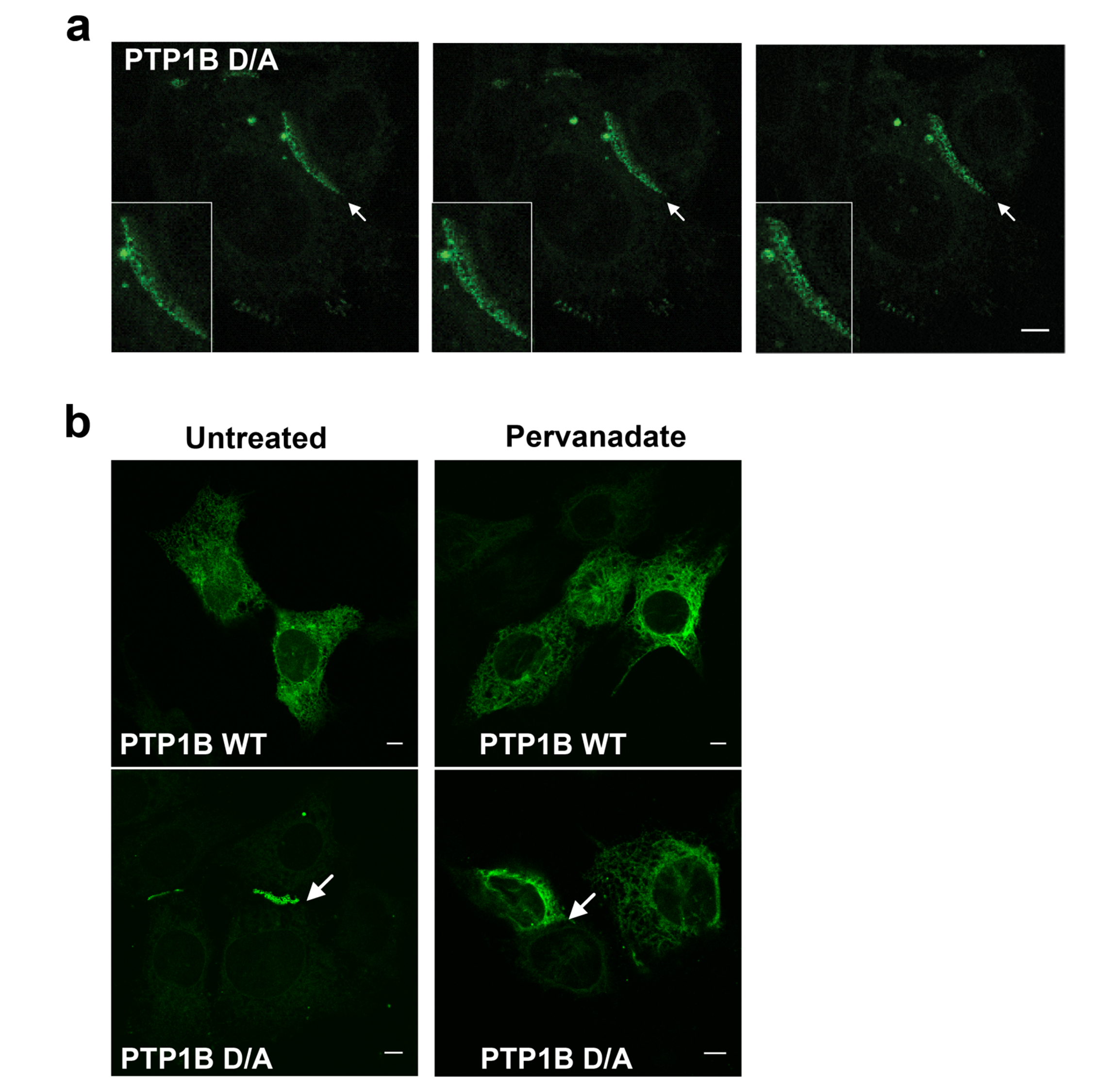

Supplement: Figure S1 — PTP1B D/A on the surface of the ER accesses PM substrates at points of cell-cell contact. (a) Serial images were acquired at successive focal planes of PTP1B-null fibroblasts expressing PTP1B D/A-GFP (Z step: 0.23 µm). Regions of cell-cell contact are indicated with arrows and magnified in the inset. Note the “honeycomb” appearance characteristic of the ER. (b) Cells expressing PTP1B WT or D/A were treated with the general tyrosine phosphatase inhibitor pervanadate to disrupt PTP-substrate interactions. Note the disappearance of PTP1B D/A localization at points of cell-cell contact after pervanadate treatment, indicating interaction of PTP1B D/A with PM bound substrates. Areas of cell-cell contact are indicated with an arrow. Scale bars correspond to 5 µm. (TIF) [file pone.0036633.s001.tif]

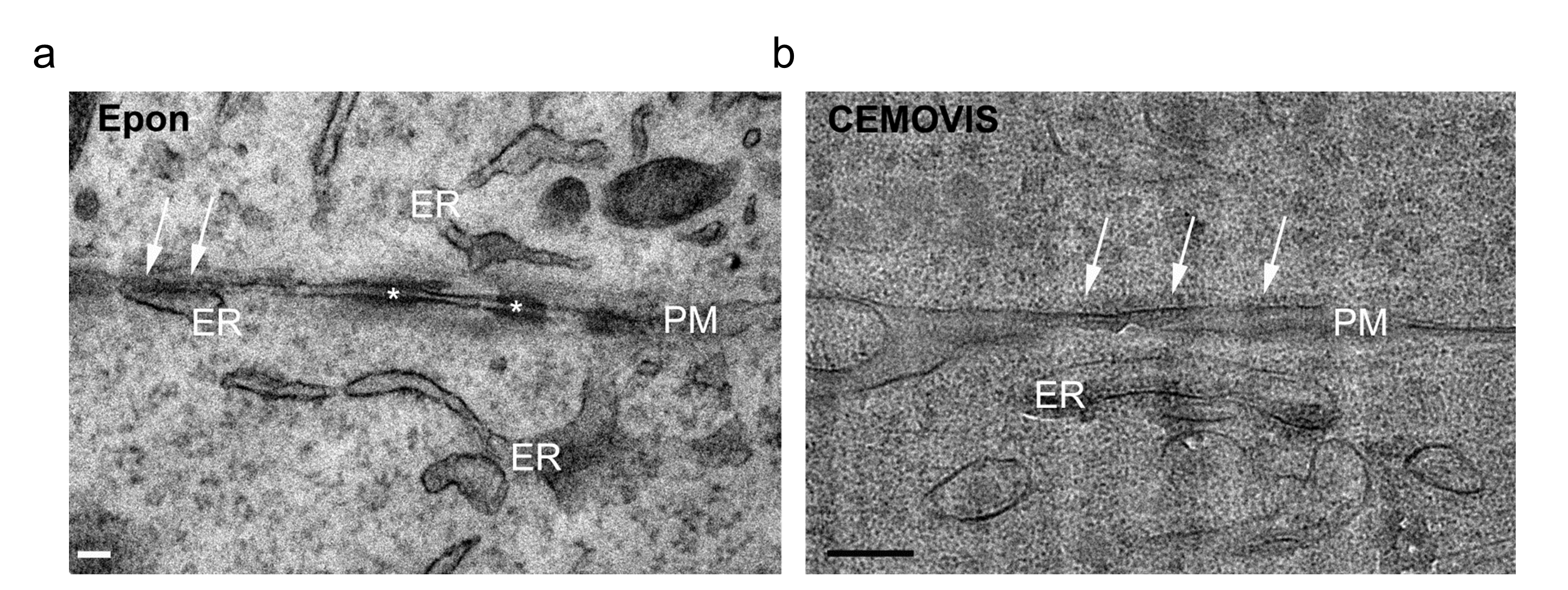

Supplement: Figure S2 — The ER also lies in proximity to the PM in HeLa cells. Epon section routine EM and high-resolution cryo-electron microscopy of vitreous sections (CEMOVIS) of wild type HeLa cells showing close association of ER and PM membranes (arrows). ER: endoplasmic reticulum; PM: plasma membranes of neighboring cells; asterisks: tight junctions; scale bar 100 nm. (TIF) [file pone.0036633.s002.tif]

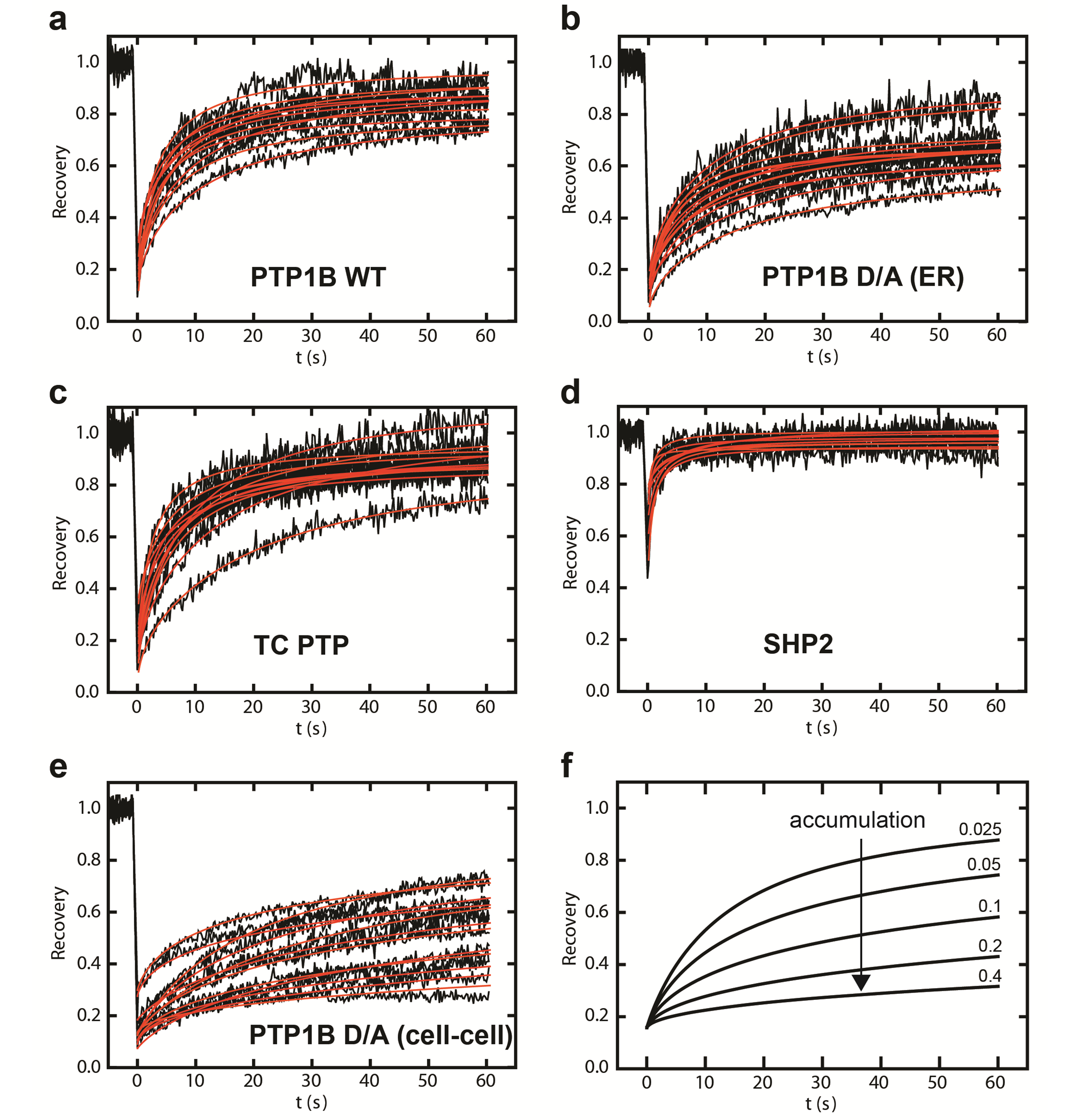

Supplement: Figure S3 — Quantitative assessment of fluorescence recovery in cells expressing various PTPs. Assessment of fluorescence recovery over time of various PTPs at the ER (a-d) or PTP1B D/A at regions of cell-cell contact (e) in different cells. The fitted model curves (red) are overlaid on the experimental data (black). (f) FRAP recovery rates of PTP1B D/A at regions of cell-cell contact in cells with different levels of PTP1B D/A accumulation. The curves correspond to different ratios of total bound to total free PTP1B D/A in the cell, which is designated as in the model (curve labels specify the values for that were used). The central curve is identical to the averaged PTP1B D/A cell-cell recovery model shown in Fig. 4b. A larger accumulation at regions of cell-cell contact clearly leads to a slower recovery. Because the level of accumulation and cell length are degenerate in the model, the exact same curves would also result by varying the cell length by similar factors. (TIF) [file pone.0036633.s003.tif]

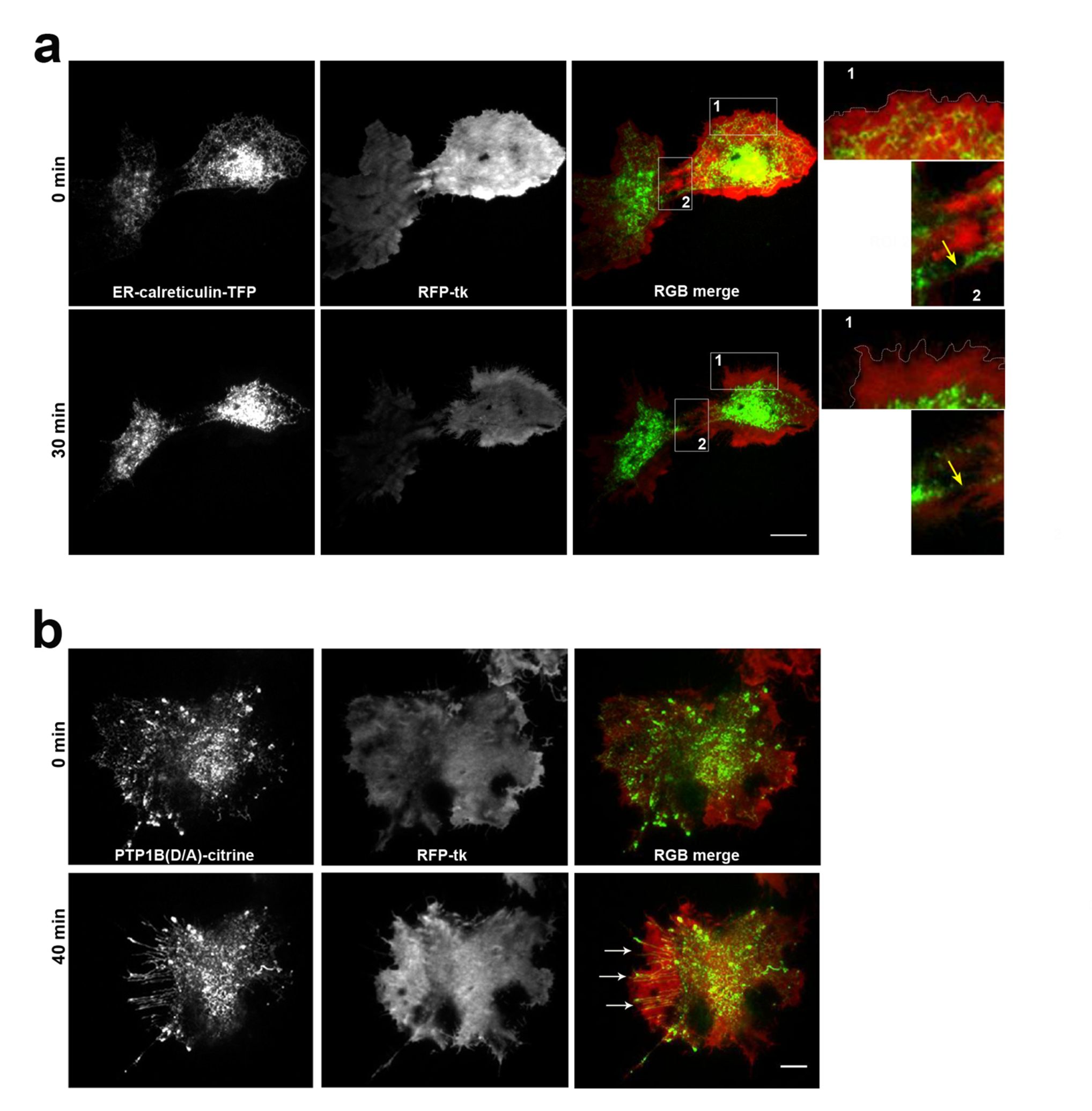

Supplement: Figure S4 — Polarity of the ER. (a) Cos-7 cells co-expressing Calreticulin-TFP and RFP-TK were treated with nocodazole (33 µM) and imaged for 30 min using TIRF microscopy. Two regions of interest “1” and “2” are highlighted and magnified in the “Merge” images. “1” shows a peripheral region of PM with no cell-cell contact; note how the ER extends out to the peripheral PM before treatment. “2” shows a region of cell-cell contact (indicated by yellow arrow); note that the ER does not retract from points of cell-cell contact after treatment. (b) Cos-7 cells co-expressing PTP1B D/A and RFP-TK were treated with nocodazole and imaged for 40 min using TIRF microscopy. In the lower right panel, arrows indicate the long stretches of ER network that remain attached to sites of adhesion to the glass coverslip following nocodazole treatment; this is observed only for cells overexpressing PTP1B D/A. Scale bars correspond to 10 µm. (TIF) [file pone.0036633.s004.tif]

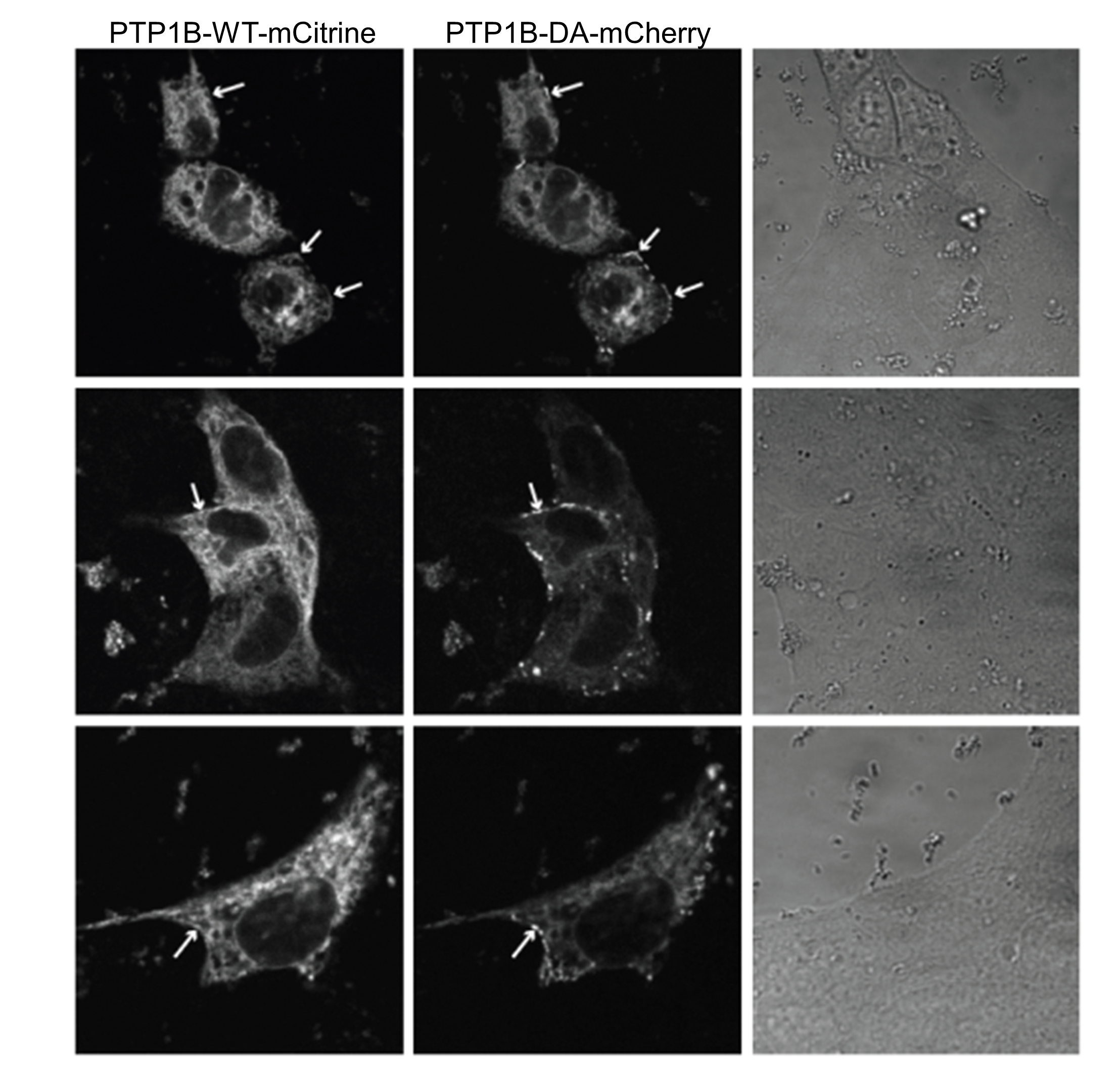

Supplement: Figure S5 — PTP1B WT localization is not altered by PTP1B D/A co-expression. Randomly growing MDCK cells were transiently co-transfected with PTP1B WT-mCitrine and PTP1B D/A-mCherry. PTP1B WT and PTP1B D/A colocalized across the entire ER; however, the D/A mutant was clearly much more highly accumulated to cell-cell contact sites. Right panel represents phase contrast images of the cells and arrows indicate sites of cell-cell contact. (TIF) [file pone.0036633.s005.tif]
